# Central corneal thickness, corneal endothelial cell density, and morphology in myopic eyes of young Chinese candidates for refractive surgery

**DOI:** 10.3389/fopht.2026.1769734

**Published:** 2026-03-06

**Authors:** Suming Wu, Zhengwei Zhang, Jing Wang, Xuechun Gong, Dongyan Guo, Nan Ye

**Affiliations:** 1 Department of Ophthalmology, Jiangnan University Medical Center, Wuxi No.2 People’s Hospital, Wuxi, Jiangsu, China; 2 Wuxi Aier Eye Hospital, Wuxi, Jiangsu, China

**Keywords:** central corneal thickness, corneal endothelial cell density, corneal endothelial cell morphology, myopia, young adults

## Abstract

**Purpose:**

To assess central corneal thickness (CCT), as well as the density and morphology of corneal endothelial cells (ECD) in myopic eyes.

**Methods:**

A total of 400 patients scheduled for myopic refractive surgery were consecutively enrolled in this study. Preoperative evaluations encompassed endothelial cell density (ECD), average cell area (CA), coefficient of variation (CV), and percentage of hexagonality (HEX), all measured using the Nidek CEM-530. Additionally, central corneal thickness (CCT) was assessed using the Pentacam HR. Ocular biometric parameters, including axial length (AL), were recorded with the IOL Master 700. Each parameter was analyzed based on ocular laterality, age, gender, spherical equivalent (SE), and AL.

**Results:**

The analysis revealed that 39% of participants were female, with a mean age of 25.36 ± 6.92 years and a mean SE of -5.06 ± 1.92D. No statistically significant differences in ECD were observed between genders, although males showed slightly higher values for HEX and CCT. No significant differences in CCT, ECD, or corneal endothelial morphology were found among subgroups categorized by SE or AL. Significant negative correlations were observed between age and both ECD (*r* = -0.197, *P* < 0.001) and CCT (*r* = -0.111, *P* = 0.026), while positive correlations were noted with CA (*r* = 0.166, *P* = 0.001) and CV (*r* = 0.364, *P* < 0.001). After adjusting for age, ECD showed no significant association with SE or AL but demonstrated a weak positive correlation with CCT (*r* = 0.128, *P* = 0.011).

**Conclusions:**

SE and AL show no significant effect on CCT or corneal ECD and morphology in our refractive surgery candidates. Weak age-CCT/ECD correlations were noted. Gender and ocular laterality effects warrant preoperative evaluation.

## Introduction

Myopia, a globally pervasive public health issue notably affecting East Asian youth, is anticipated to exceed 740 million cases worldwide by 2050 (1). Despite the escalating prevalence and severity of myopia, current clinical strategies lack fully effective, evidence-based interventions to comprehensively prevent its onset and progression (2). With advancing surgical techniques and growing demand for spectacle-free lifestyles, refractive procedures are experiencing rising adoption. In recent decades, phakic intraocular lens (pIOL) implantation has emerged as a viable therapeutic strategy within refractive surgical protocols, particularly for young adults requiring correction of high myopia, in whom deep excimer laser stromal ablation—necessary for large refractive errors—would increase the risk of postoperative corneal ectasia. However, growing concerns have been raised regarding the long-term safety of iris-claw pIOLs, particularly due to progressive endothelial cell loss (3, 4). Consequently, posterior chamber pIOLs appear to be the more favorable option (5).

While current evidence suggests minimal corneal endothelial impact from laser keratorefractive surgery (6), isolated cases of corneal decompensation have been reported in patients with preexisting endothelial pathology, such as Fuchs endothelial corneal dystrophy (FECD), manifested as central corneal guttata. For example, one case involved a 58-year-old woman with preoperative FECD who developed persistent corneal edema and vision loss following laser-assisted *in situ* keratomileusis (LASIK) (7), while another described a 47-year-old woman with asymptomatic guttata who experienced bilateral corneal decompensation post-LASIK (8). Additionally, Moshirfar et al. (9) reported a 12.4% decrease in endothelial cell density (ECD) and increased central corneal thickness one year after LASIK in eyes with corneal guttata. These findings underscore the importance of careful patient selection, as individuals with compromised endothelium may not be suitable candidates for laser vision correction. In light of the above, assessing endothelial morphometric parameters remains critical for risk stratification in pIOL implantation in young adults (10). Therefore, refractive surgeons should incorporate perioperative evaluation of endothelial functional integrity in all pIOL candidates and in keratorefractive surgery candidates who exhibit any abnormal endothelial findings on slit-lamp examination.

Although prior investigations have characterized corneal endothelial cell density and morphology in Chinese pediatric populations (11–13) and individuals over 40 years of age, both with or without myopia (14–16), a critical knowledge gap persists regarding corneal endothelial parameters and central corneal thickness (CCT) in myopic young Chinese adults, who constitute the predominant candidates undergoing refractive surgery. This retrospective cross-sectional study involved young myopic patients scheduled for refractive surgery at our hospital. During preoperative evaluations, we systematically gathered demographic data and ocular parameters. The findings offer valuable and detailed insights into the corneal characteristics of this surgical group.

## Methods

### Study design

This retrospective cross-sectional study was carried out at Wuxi Aier Eye Hospital, located in Jiangsu Province, China. Written informed consent was obtained from all participants prior to their inclusion in the study. The research adhered to the principles outlined in the Declaration of Helsinki and complied with the ARVO statement regarding human subjects. The study protocol received approval from the Institutional Review Board of Wuxi Aier Eye Hospital (Approval No. WXAE-ETH-2024-018).

### Population and sample

We recruited candidates for refractive surgery from Wuxi Aier Eye Hospital, China, who were of Han Chinese ethnicity and aged 18 years or older. The inclusion criteria were as follows: (1) comprehensive preoperative ocular and systemic evaluations were completed; (2) spherical equivalent (SE) refractive error of ≤ -0.50D in each eye; and (3) best-corrected visual acuity (BCVA) of ≥ 20/20 in each eye. Exclusion criteria included: (1) anatomical abnormalities of the eye (such as corneal or ocular surface diseases, glaucoma, or uveitis); (2) a history of inflammation or infection (including active or past ocular infections); (3) a history of surgery or trauma (such as ocular trauma or prior intraocular or corneal procedures); (4) external factors that could interfere with results (e.g., contact lens usage within the past month or recent use of ocular medication); and (5) systemic diseases (including diabetes mellitus, autoimmune disorders, and others). When only one eye’s data from each participant was needed for statistical analysis, we used data from the right eyes to minimize potential confounding effects from high interocular correlation.

### Data collection procedures

At the baseline assessment, all subjects underwent standardized preoperative evaluations, including comprehensive systemic and ocular examinations. Ocular biometric parameters were measured, with axial length (AL) assessed using the IOL Master 700 (Carl Zeiss Meditec AG, Germany; Ver. 1.90R38), and intraocular pressure (IOP) determined using the Nidek NT-510 tonometer (Nidek Co., Ltd., Japan). Corneal endothelial analysis was performed with the Nidek CEM-530 specular microscope (Nidek Co., Ltd., Japan), capturing parameters such as endothelial cell density (ECD), coefficient of variation (CV), average cell area (CA), and hexagonality percentage (HEX). Only sharply defined images, where the corneal endothelial cells were automatically delineated and analyzed, were deemed valid for examination. Additionally, central corneal thickness (CCT) and pupillary diameter were obtained using Scheimpflug tomography through the Pentacam HR system (Oculus Optikgeräte GmbH, Germany; Ver. 1.26r28) in a dimly lit examination room. The corneas with a CCT < 500 μm were screened for keratoconus using anterior and posterior elevation maps. All imaging procedures were carried out by a certified ophthalmic imaging specialist, adhering strictly to standardized protocols.

### Data analysis

Continuous variables were expressed as the mean ± standard deviation (SD). The normality of their distributions was assessed using the one-sample Kolmogorov-Smirnov test prior to applying tests of significance. All continuous variables exhibited normal distributions, with the exceptions of age and IOP. Furthermore, the continuous variables refractive state and AL were categorized into subgroups. Refraction subgroups were stratified based on SE as follows: low myopia (-3.0D < SE ≤ -0.50D), moderate myopia (-6.0D < SE ≤ -3.0D), and high myopia (SE ≤ -6.0D). AL subgroups were classified as medium eyes (AL < 24.0 mm), medium-long eyes (24.0 mm ≤ AL < 26.0 mm), and long eyes (AL ≥ 26.0 mm). Besides, the participants were stratified into two age-based groups: those under 30 years of age and those aged 30 years or older. Besides, given the significant age difference between the male and female groups, analysis of covariance (ANCOVA) with age as a covariate was used to compare corneal parameters between genders.

A paired t-test was performed to compare parameters between the right and left eyes, while an independent Student’s t-test or Mann–Whitney U test was used to examine differences in continuous variables between age and gender groups, based on the normality of data distribution. One-way analysis of variance (ANOVA) with *post hoc* Bonferroni correction was conducted to compare examination parameters among patients grouped by SE and AL subgroups. The correlation between age and corneal parameters was analyzed using Spearman’s correlation coefficient. Associations between ocular parameters (SE or AL) and corneal metrics, including CCT and corneal endothelial characteristics (ECD, CV, CA, and HEX), were evaluated using Pearson’s correlation coefficient, with adjustments for age and gender.

Univariate linear regression was employed to assess the significance of associations between ECD and continuous variables: age, IOP, SE, AL, WTW, and CCT. Variables demonstrating significant associations (*P* < 0.05) with ECD were subsequently included in a multiple linear regression model. All statistical analyses were performed using SPSS 21.0 (SPSS Inc., Chicago, Illinois, USA), and statistical significance was defined as *P* < 0.05.

## Results

The study included 400 participants (156 females and 244 males) with a mean age of 25.4 ± 6.9 years (range: 18–43 years). **Table 1** details the demographic data and preoperative binocular status of the study population. The right eye demonstrated greater scotopic pupillary diameter, IOP, AL, and CA. In contrast, the left eye showed superior ECD, and CCT. Interocular comparisons revealed no statistically significant disparities in anterior chamber depth (ACD), lens thickness (LT), white-to-white measurement (WTW), CV, or HEX.

**Table 1:** Demographic data and binocular status of the 400 preoperative myopic patients.

Parameters	All eyes	OD	OS	*P*
No.	800	400	400	
Pupil diameter (mm)	3.42 ± 0.68 (1.93-5.92)	3.53 ± 0.71 (1.93-5.92)	3.32 ± 0.66 (1.97-5.47)	**<0.001**
IOP (mmHg)	15.42 ± 2.85 (8.0-25.0)	15.54 ± 2.88 (9.0-24.0)	15.29 ± 2.82 (8.0-25.0)	**0.001**
SE (D)	-4.92 ± 1.91 (-15.37--0.50)	-5.06 ± 1.92 (-15.37--0.50)	-4.78 ± 1.89 (-10.63--0.50)	**<0.001**
AL (mm)	25.71 ± 1.19 (22.10-37.54)	25.77 ± 1.26 (22.98-37.54)	25.64 ± 1.12 (22.10-29.53)	**0.001**
ACD (mm)	3.72 ± 0.27 (2.78-4.52)	3.72 ± 0.27 (2.78-4.52)	3.72 ± 0.26 (3.08-4.49)	0.122
LT (mm)	3.62 ± 0.28 (2.90-5.21)	3.62 ± 0.28 (2.90-5.21)	3.63 ± 0.27 (2.90-4.62)	0.779
WTW (mm)	12.08 ± 0.41 (10.40-13.50)	12.08 ± 0.40 (11.1-13.3)	12.08 ± 0.42 (10.4-13.5)	0.948
ECD (cells/mm^2^)	2993.50 ± 236.18 (2113-3991)	2982.39 ± 227.22 (2261-3991)	3004.61 ± 244.58 (2113-3988)	**0.005**
CA (µm^2^)	317.99 ± 25.90 (249.0-455.0)	318.97 ± 25.03 (265.0-425.0)	317.01 ± 26.73 (249.0-455.0)	**0.006**
CV (%)	28.53 ± 4.85 (19.0-75.0)	28.50 ± 4.49 (19.0-47.0)	28.57 ± 5.19 (20.0-75.0)	0.774
HEX (%)	64.94 ± 5.90 (26-82)	65.14 ± 5.83 (29.0-82.0)	64.75 ± 5.97 (26.0-80.0)	0.209
CCT (μm)	548.41 ± 29.00 (468-629)	547.72 ± 29.01 (468.0-629.0)	549.10 ± 29.00 (477.0-629.0)	**<0.001**

OD, right eye; OS, left eye; IOP, intraocular pressure; SE, spherical equivalent; AL, axial length; LT, lens thickness; WTW, white to white; ECD, endothelial cell density; CA, average cell area; CV, coefficient of variation; HEX, percent of hexagonality; CCT, central corneal thickness.

‌Bold‌ values indicate statistically significant differences.

The mean values of the measured parameters, categorized by gender, are presented in **Table 2**. No statistically significant differences were found between genders in SE, CA, or ECD. However, notable gender-based variations were observed in several other parameters. Males showed higher values for AL, ACD, WTW, HEX, and CCT, whereas females exhibited greater values for LT and CV.

**Table 2:** Demographic data and right eye status of the 400 participants.

Parameters	All eyes (OD)	Female	Male	*P*
No.	400	156	244	
Age	25.36 ± 6.92 (18-43)	29.39 ± 6.95 (18-43)	22.78 ± 5.55 (18-40)	**<0.001**
Pupil diameter (mm)	3.53 ± 0.71 (1.93-5.92)	3.40 ± 0.70 (2-5.34)	3.60 ± 0.70 (1.93-5.92)	**0.006**
IOP (mmHg)	15.54 ± 2.88 (9.0-24.0)	14.89 ± 2.47 (9-20)	15.96 ± 3.04 (9-24)	**<0.001**
SE (D)	-5.06 ± 1.92 (-15.38--0.50)	-5.03 ± 1.63 (-9.50--0.50)	-5.08 ± 2.08 (-15.38--0.50)	0.777
AL (mm)	25.77 ± 1.26 (22.98-37.54)	25.31 ± 1.00 (22.98-27.63)	26.06 ± 1.32 (23.43-37.54)	**<0.001**
ACD (mm)	3.72 ± 0.27 (2.78-4.52)	3.60 ± 0.25 (3.07-4.27)	3.80 ± 0.25 (2.78-4.52)	**<0.001**
LT (mm)	3.62 ± 0.28 (2.90-5.21)	3.74 ± 0.28 (2.90-4.59)	3.54 ± 0.24 (2.97-5.21)	**<0.001**
WTW (mm)	12.08 ± 0.40 (11.1-13.3)	11.94 ± 0.38 (11.1-12.9)	12.17 ± 0.39 (11.2-13.3)	**<0.001**
ECD (cells/mm^2^)	2982.39 ± 227.22 (2261-3991)	2971.06 ± 251.97 (2263-3991)	2989.64 ± 210.10 (2261-3563)	0.122*
CA (µm^2^)	318.97 ± 25.03 (265.0-425.0)	319.74 ± 25.95 (265.0-425.0)	318.48 ± 24.47 (267-418)	0.428*
CV (%)	28.50 ± 4.49 (19.0-47.0)	30.06 ± 4.74 (21.0-42.0)	27.50 ± 4.02 (19.0-47.0)	0.006*
HEX (%)	65.14 ± 5.83 (29-82)	64.20 ± 5.91 (48.0-82.0)	65.74 ± 5.71 (29.0-78.0)	0.251*
CCT (μm)	547.72 ± 29.01 (468-629)	543.62 ± 28.57 (477-629)	550.34 ± 29.05 (468-614)	0.031*

OD, right eye; IOP, intraocular pressure; SE, spherical equivalent; AL, axial length; LT, lens thickness; WTW, white to white; ECD, endothelial cell density; CA, average cell area; CV, coefficient of variation; HEX, percent of hexagonality; CCT, central corneal thickness. * analysis of covariance (ANCOVA) adjusting for age.

Bold values indicate statistically significant differences.

In **Table 3**, the subjects were classified into three groups based on AL: 22 medium eyes (AL < 24.0 mm), 220 medium-long eyes (24.0 mm ≤AL < 26.0 mm), and 158 long eyes (AL ≥ 26.0 mm). No statistically significant differences were detected in ECD or morphology parameters (CV, CA, HEX), or CCT.

**Table 3:** Baseline and corneal endothelial cell characteristics among different axial length groups.

Parameters	All eyes (OD)	AL<24.0mm	24.0mm≤AL<26mm	AL≥26.0mm	*P*
No.	400	22	220	158	
Age	25.36 ± 6.92 (18-43)	27.86 ± 7.29 (18-43)	26.08 ± 7.17 (18-43)	24.00 ± 6.29 (18-43)	**0.003**
Pupil diameter (mm)	3.57 ± 0.70 (1.93-5.92)	3.46 ± 0.65 (2.65-4.50)	3.50 ± 0.70 (2.00-5.92)	3.57 ± 0.72 (1.93-5.45)	0.560
IOP (mmHg)	15.54 ± 2.88 (9.0-24.0)	15.36 ± 2.85 (10.0-21.0)	15.52 ± 3.01 (9.0-24.0)	15.59 ± 2.70 (9.0-23.0)	0.929
SE (D)	-5.06 ± 1.92 (-15.37--0.5)	-2.70 ± 1.72 (-5.25--0.5)	-4.44 ± 1.61 (-15.37--1.50)	-6.26 ± 1.60 (-11.00--1.75)	**<0.001**
AL (mm)	25.77 ± 1.26 (22.98-37.4)	23.61 ± 0.27 (22.98-23.95)	25.17 ± 0.51 (24.01-25.99)	26.91 ± 1.10 (26.00-37.54)	**<0.001**
ACD (mm)	3.72 ± 0.27 (2.78-4.52)	3.50 ± 0.26 (3.07-4.32)	3.67 ± 0.25 (3.09-4.33)	3.82 ± 0.26 (2.78-4.52)	**<0.001**
LT (mm)	3.62 ± 0.28 (2.90-5.21)	3.78 ± 0.26 (3.32-4.37)	3.64 ± 0.27 (3.09-4.59)	3.56 ± 0.29 (2.90-5.21)	**0.001**
WTW (mm)	12.08 ± 0.40 (11.1-13.3)	11.80 ± 0.40 (11.2-12.6)	12.01 ± 0.39 (11.1-11.3)	12.22 ± 0.37 (11.4-13.1)	**<0.001**
ECD (cells/mm^2^)	2982.39 ± 227.22 (2261-3991)	2955.45 ± 189.65 (2639-3273)	2984.42 ± 218.72 (2261-3991)	2983.32 ± 244.04 (2263-3904)	0.849
CA (µm^2^)	318.97 ± 25.03 (265.0-425.0)	321.96 ± 21.85 (282.0-364.0)	317.74 ± 23.87 (267.0-418.0)	320.27 ± 26.99 (265.0-425.0)	0.532
CV (%)	28.50 ± 4.49 (19.0-47.0)	27.91 ± 4.89 (21.0 -41.0)	28.82 ± 4.43 (19.0-42.0)	28.15 ± 4.51 (20.0-47.0)	0.291
HEX (%)	65.14 ± 5.83 (29.0-82.0)	66.36 ± 5.86 (57.0-79.0)	64.63 ± 5.76 (48.0-82.0)	65.67 ± 5.89 (29.0-77.0)	0.143
CCT (μm)	547.72 ± 29.01 (468.0-629.0)	539.34 ± 27.16 (481.0-606.0)	548.26 ± 30.50 (468.0-629.0)	550.13 ± 26.40 (493.0-614.0)	0.095

OD, right eye; IOP, intraocular pressure; SE, spherical equivalent; AL, axial length; LT, lens thickness; WTW, white to white; ECD, endothelial cell density; CA, average cell area; CV, coefficient of variation; HEX, percent of hexagonality; CCT, central corneal thickness.

Bold‌ values indicate statistically significant differences.

In **Table 4**, participants were stratified into three refractive groups based on SE: low-myopia (-3.0D<SE≤-0.50D, n=50), moderate-myopia (-6.0D<SE≤-3.0D, n=220), and high-myopia (SE ≤ -6.0 D, n=158). Similar to AL-based grouping, no statistically significant differences were detected in ECD or morphology parameters (CV, CA, HEX), or CCT.

**Table 4:** Baseline and corneal endothelial cell characteristics among different spherical equivalent groups. .

Parameters	All eyes (OD)	-3.0D<SE≤-0.50D	-6.0D<SE≤-3.0D	SE≤-6.0D	*P*
No.	400	50	228	122	
Age	25.36 ± 6.92 (18.0-43.0)	26.08 ± 7.55 (18.0-41.0)	25.43 ± 6.90 (18.0-43.0)	24.91 ± 6.72 (18.0-43.0)	0.583
Pupil diameter (mm)	3.53 ± 0.71 (1.93-5.92)	3.44 ± 0.63 (2.36-4.85)	3.52 ± 0.68 (1.93-5.45)	3.57 ± 0.78 (2.00-5.92)	0.545
IOP (mmHg)	15.54 ± 2.88 (9.0-24.0)	15.64 ± 3.16 (10.0-22.0)	15.40 ± 2.92 (9.0-24.0)	15.77 ± 2.68 (9.0-23.0)	0.491
SE (D)	-5.06 ± 1.92 (-15.37--0.50)	-2.11 ± 0.72 (-2.87--0.50)	-4.53 ± 0.83 (-5.87--3.00)	-7.26 ± 1.27 (-15.37--6.00)	**<0.001**
AL (mm)	25.77 ± 1.26 (22.98-37.54)	24.57 ± 0.75 (22.98-26.30)	25.57 ± 1.19 (23.18-37.54)	26.63 ± 0.98 (24.38-29.94)	**<0.001**
ACD (mm)	3.72 ± 0.27 (2.78-4.52)	3.67 ± 0.23 (3.28-4.32)	3.71 ± 0.26 (3.07-4.33)	3.76 ± 0.29 (2.78-4.52)	0.104
LT (mm)	3.62 ± 0.28 (2.90-5.21)	3.65 ± 0.28 (3.09-4.37)	3.62 ± 0.28 (2.90-4.59)	3.61 ± 0.31 (3.09-5.21)	0.761
WTW (mm)	12.08 ± 0.40 (11.1-13.3)	12.13 ± 0.43 (11.2-13.2)	12.07 ± 0.40 (11.1-13.3)	12.09 ± 0.38 (11.4-12.9)	0.607
ECD (cells/mm^2^)	2982.39 ± 227.22 (2261-3991)	2991.22 ± 252.89 (2261-3991)	2969.10 ± 213.86 (2263-3563)	3003.62 ± 240.26 (2405-3904)	0.384
CA (µm^2^)	318.97 ± 25.03 (265.0-425.0)	319.34 ± 25.43 (267.0-418.0)	319.88 ± 24.95 (266.0-425.0)	317.12 ± 25.14 (265.0-402.0)	0.613
CV (%)	28.50 ± 4.49 (19.0-47.0)	28.06 ± 4.98 (19.0-41.0)	28.34 ± 4.07 (21.0-39.0)	28.99 ± 4.99 (20.0-47.0)	0.327
HEX (%)	65.14 ± 5.83 (29.0-82.0)	65.84 ± 6.16 (51.0-79.0)	65.25 ± 5.40 (53.0-82.0)	64.64 ± 6.45 (29.0-76.0)	0.428
CCT (μm)	547.72 ± 29.01 (468.0-629.0)	539.34 ± 27.16 (481.0-606.0)	548.26 ± 30.50 (468.0-629.0)	550.13 ± 26.40 (493.0-614.0)	0.078

OD, right eye; IOP, intraocular pressure; SE, spherical equivalent; AL, axial length; LT, lens thickness; WTW, white to white; ECD, endothelial cell density; CA, average cell area; CV, coefficient of variation; HEX, percent of hexagonality; CCT, central corneal thickness.

Bold values indicate statistically significant differences.

In **Table 5**, participants were stratified by age into two groups: <30 years and ≥30 years. No statistically significant differences were observed between the groups in SE, HEX, or CCT. The younger group exhibited higher ECD values but lower CA and CV values.

**Table 5:** Baseline and corneal endothelial cell characteristics between different age groups. .

Parameters	All eyes (OD)	< 30 years	≥30 years	*P*
No.	400	283	117	
Age	25.36 ± 6.92 (18-43)	21.50 ± 3.23 (18-29)	34.83 ± 3.23 (30-43)	**<0.001**
Pupil diameter (mm)	3.53 ± 0.71 (1.93-5.92)	3.56 ± 0.69 (2.00-5.92)	3.45 ± 0.74 (1.93-5.45)	0.168
IOP (mmHg)	15.54 ± 2.88 (9.0-24.0)	15.87 ± 2.94 (9.0-24.0)	14.74 ± 2.54 (10.0-21.0)	**<0.001**
SE (D)	-5.06 ± 1.92 (-15.38--0.50)	-5.14 ± 1.94 (-11.0--0.50)	-4.86 ± 1.86 (-15.38--1.63)	0.183
AL (mm)	25.77 ± 1.26 (22.98-37.54)	25.96 ± 1.32 (23.18-37.54)	25.31 ± 0.98 (22.98-27.63)	**<0.001**
ACD (mm)	3.72 ± 0.27 (2.78-4.52)	3.79 ± 0.25 (3.07-4.52)	3.57 ± 0.25 (2.78-4.09)	**<0.001**
LT (mm)	3.62 ± 0.28 (2.90-5.21)	3.51 ± 0.20 (2.90-4.01)	3.89 ± 0.28 (3.32-5.21)	**<0.001**
WTW (mm)	12.08 ± 0.40 (11.1-13.3)	12.14 ± 0.39 (11.2-13.3)	11.95 ± 0.39 (11.1-13.0)	**<0.001**
ECD (cells/mm^2^)	2982.39 ± 227.22 (2261-3991)	3007.51 ± 214.20 (2405-3904)	2921.63 ± 246.53 (2261-3991)	**0.001**
CA (µm^2^)	318.97 ± 25.03 (265.0-425.0)	315.98 ± 23.44 (265-402)	326.21 ± 27.30 (269-425)	**<0.001**
CV (%)	28.50 ± 4.49 (19.0-47.0)	27.81 ± 4.40 (19.0-47.0)	30.19 ± 4.28 (21.0-42.0)	**<0.001**
HEX (%)	65.14 ± 5.83 (29-82)	65.42 ± 5.81 (48-82)	64.44 ± 5.85 (29-75)	0.127
CCT (μm)	547.72 ± 29.01 (468-629)	548.97 ± 30.18 (468-629)	544.69 ± 25.86 (485-606)	0.181

OD, right eye; IOP, intraocular pressure; SE, spherical equivalent; AL, axial length; LT, lens thickness; WTW, white to white; ECD, endothelial cell density; CA, average cell area; CV, coefficient of variation; HEX, percent of hexagonality; CCT, central corneal thickness.

Bold values indicate statistically significant differences.

**Figure 1:**
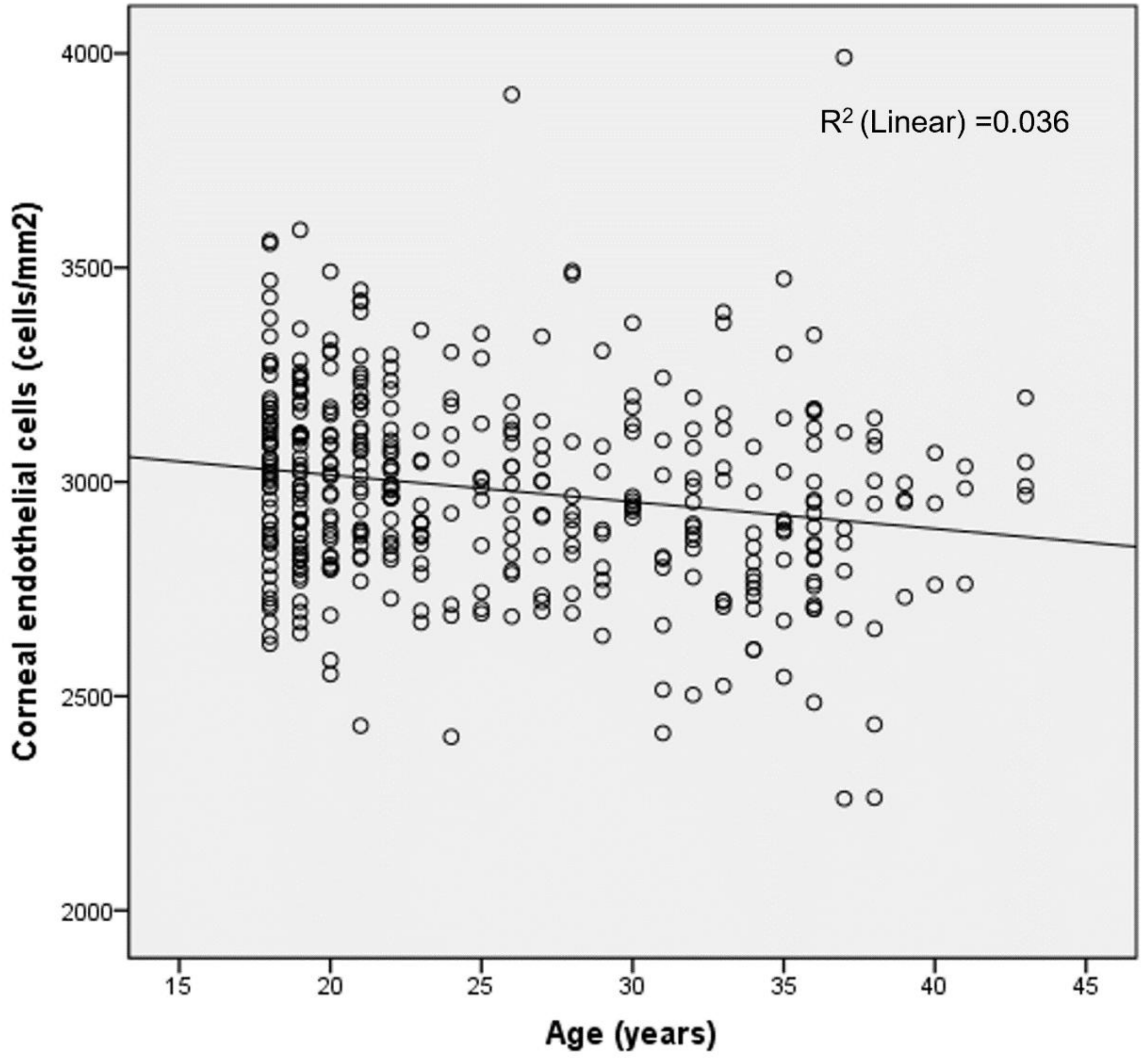
Scatter plot showing a significant negative correlation between endothelial cell density (ECD) and age (*r* = -0.197, *P* < 0.001). Univariable linear regression analysis yielded the equation: ECD = 3141.91+(-6.281*Age).

Significant negative correlations were observed between age and both ECD (*r* = -0.197, *P* < 0.001, **Figure 1**) and CCT (*r* = -0.111, *P* = 0.026), whereas positive correlations were identified with CA (*r* = 0.166, *P* = 0.001) and CV (*r* = 0.364, *P* < 0.001). Univariable linear regression analysis derived the following regression equation between ECD and age: ECD = 3141.91+(-6.281*age). After adjusting for age, ECD showed no significant association with SE (*P* = 0.662) or AL (*P* = 0.179) but exhibited a weak positive correlation with CCT (*r* = 0.128, *P* = 0.011).

**Table 6** presents the results of both univariable and multivariable linear regression models for ECD. Four variables that showed a significant association with ECD in the univariable analysis were subsequently included in the multivariable model. In the multivariable linear regression analysis, three variables—age, WTW, and CCT—remained significantly associated with ECD.

**Table 6:** The univariate and multivariate linear regression models for corneal endothelial cell density.

Variables	Univariate linear regression			Multivariate linear regression		
	B	95% CI	*P*	B	95% CI	*P*
Age	-6.28	-9.48, -3.09	<0.001	-4.66	-8.00, -1.33	0.006
WTW	88.02	32.38, 143.65	0.002	71.88	12.04, 131.72	0.019
CCT	1.12	0.35, 1.88	0.004	1.09	0.32, 1.87	0.006
AL	19.80	2.16, 37.43	0.028			
SE	-3.42	-15.10, 8.26	0.566			
IOP	-5.61	-13.38, 2.16	0.157			

WTW, white to white; CCT, central corneal thickness; AL, axial length; SE, spherical equivalent; IOP, intraocular pressure.

## Discussion

The evaluation of CCT, a key reference index for corneal refractive surgery, and ECD and morphology, critical indicators for intraocular refractive surgery, forms an essential component of refractive surgical practice. These parameters are pivotal for preoperative assessment and postoperative monitoring. Contemporary evidence, as delineated in a recent review, emphasizes that CCT modulation is governed by a multifactorial interplay of determinants, encompassing demographic variables (e.g., age, gender, and ethnicity), systemic comorbidities such as autoimmune diseases, and extrinsic environmental influences (17). As a result, the corneal parameter profiles of various populations undergoing refractive surgery merit further exploration. This study specifically examined young myopic patients of Han Chinese ethnicity residing in Wuxi, China.

Axial elongation in myopia may lead to “stretching” of ocular tissues, potentially including the corneal endothelium, which could result in decreased cell density or altered morphology. In the present study, no statistically significant differences in CCT or ECD and corneal endothelial morphology (CV, CA, HEX) were observed between subgroups categorized by AL or SE (**Tables 3**, **4**). In comparison, Zhang et al. (16) recently reported that highly myopic eyes (axial length: 29.09 ± 2.33 mm) exhibited higher ECD (2730.76 ± 259.03 cells/mm^2^ vs. 2677.74 ± 240.93 cells/mm^2^) but greater pleomorphism and polymegathism compared to controls (axial length: 23.55 ± 1.00 mm) in an elderly cohort (mean age: 65.98 ± 9.83 years). Notably, they found no statistically significant differences in CCT between intergroup comparisons. Panda et al. (18) recently also reported no significant intergroup differences in CCT across 100 myopic, hyperopic, and emmetropic eyes, with no correlation observed between CCT and AL. These findings align with prior studies analyzing data from 150 right eyes of healthy young Egyptian adults (aged 20–30 years) (19) and 111 right eyes of myopic Chinese-Malaysian pediatric subjects (aged 8–9 years) (12).

However, Hafeez et al. (20) recently observed in their analysis of 129 myopic patients (aged 14–40 years) that ECD exhibited a more pronounced reduction in individuals with high myopia compared to those with low-to-moderate myopia. Interestingly, Mutwaly et al. (21) reported a graded relationship between myopia severity and CCT in a young Sudanese cohort (N = 160; aged 16–38 years), with CCT measurements decreasing progressively from low to high myopia, whereas ECD showed no significant intergroup variation across all myopia subgroups. While the associations between CCT or ECD and ametropia or axial length vary across studies, their relationship with age remains remarkably consistent in the literature. These findings further demonstrate that age serves as the primary determinant of ECD reduction in healthy population (22), while refractive error and axial elongation exert negligible effects on ECD or CCT. These results also support the notion that the anterior segment remains relatively stable during the process of ocular elongation in myopia (23). A progressive decline in ECD with advancing age was paralleled by corresponding reductions in CCT (14, 15, 24, 25), as corroborated by the observations in our study (**Table 6**).

Interestingly, we observed a significant association between WTW diameter and ECD in both univariable and multivariable linear regression models (**Table 6**). The theoretical basis for this association may stem from the embryological and anatomical relationship between corneal diameter and overall ocular dimensions. Larger WTW measurements typically correlate with greater corneal diameter. Given that ECD represents cell count per unit area, eyes with larger overall dimensions may exhibit distinct endothelial characteristics due to differential tangential stretching during ocular growth and development. Furthermore, WTW serves as a proxy for anterior segment size (26), which may influence endothelial cell distribution patterns and metabolic demands. Notably, WTW emerged as a significant independent predictor even after adjusting for age and CCT, suggesting a genuine anatomical association that warrants further investigation.

It is noteworthy that, while most studies have found no significant correlation between the degree of myopia or AL and ECD, the likelihood of morphological abnormalities in the corneal endothelium—such as guttata—tends to increase with higher degrees of myopia in both younger (21) and old populations (16). Consequently, when assessing ECD values, particular attention should be given to the morphological characteristics of the corneal endothelium, particularly in patients with high myopia or elongated eyes. The presence of guttata is a finding that can occasionally be observed in specular microscopy of young adults. The default software in currently available specular microscopy devices is not capable of automatically identifying guttata, as these systems do not yet incorporate artificial intelligence (AI) algorithms capable of labeling them (27). In the present study, images were not reviewed to confirm the absence of guttata, so this finding may have been missed in a number of cases (likely small), which could have had some impact on the results and represents a limitation of this study. Given the potential risks associated with undetected guttata, particularly in the context of post-refractive surgery corneal decompensation, we strongly recommend the incorporation of AI-assisted identification methods in future clinical protocols.

Additionally, young and middle-aged adults with refractive errors represent a key demographic for the use of corneal contact lenses. Although our study did not specifically investigate the effects of contact lens use on corneal parameters within the examined cohort, findings from previous research provide significant insights. Two recent studies have independently explored the impact of hard contact lenses (HCL) and soft contact lenses (SCL) on corneal endothelial cell density and morphology (28, 29). Ono et al. (28) revealed that prolonged use of HCL (>14 years) induces morphological alterations in the corneal endothelium, including polymegathism (reflected by increased CV) and polymorphism (evidenced by reduced HEX), while ECD remains unaffected. Furthermore, a subsequent study by Ono et al. (29) demonstrated that prolonged SCL wear (>1 year) induces alterations in both corneal endothelial morphology (elevated CV, diminished HEX) and reduced ECD. Given these findings, clinicians are advised to perform comprehensive baseline evaluations of corneal endothelial cell morphology and density before fitting contact lenses. Regular monitoring of endothelial integrity is essential for patients with a history of long-term use of both HCL and SCL (30). These results underscore the importance of meticulous preoperative assessment for individuals who have worn contact lenses extensively, especially in the context of refractive surgery. Particular attention should be directed toward evaluating ECD and morphological changes in this patient population.

Although this study primarily incorporates corneal data from the right eyes for final statistical analysis, the symmetry of corneal parameters between both eyes is also noteworthy. Our investigation revealed statistically significant increases in left-eye CCT (OS: 549.10 ± 29.00 μm vs. OD: 547.72 ± 29.01 μm; *P* < 0.001) and ECD (OS: 3004.61 ± 244.58 cells/mm^2^ vs. OD: 2982.39 ± 227.22 cells/mm^2^; *P* = 0.005), without significant interocular disparities in endothelial morphology (CV: *P* = 0.774; HEX: *P* = 0.209). Our findings align with a prior multicenter study involving 6,644 Chinese myopic patients (mean age 25.12 ± 5.44 years) (31) and a population-based study involving 5792 Russian subjects (mean age 58.8 ± 10.6 years) (32), which similarly reported significantly greater CCT in right eyes compared to right eyes. However, Kelekele et al. (33) observed no statistically significant interocular variations in ECD, HEX, or CCT parameters within their Congolese cohort (N = 278; mean age 38.9 ± 17.2 years). This absence of laterality was echoed in a Pakistani population study (N = 362; mean age 38.3 ± 10.2 years) by Shaikh and associates (25). These variations may reflect differences in sample size and demographic heterogeneity, particularly variations in racial composition. Although the interocular differences of CCT and ECD observed in our study are indeed statistically significant yet clinically negligible, the findings emphasize the importance of accounting for interocular corneal parameter differences when designing and interpreting large-scale population studies.

Ethnicity is undeniably an important clinical factor that may influence CCT or ECD. When comparing the CCT (547.72 ± 29.01 μm) and ECD (2982.39 ± 227.22 cells/mm²) values observed in our study with those reported in studies involving similar age groups (18–43 years), our findings are consistent with those of a study conducted on a larger cohort of young myopic patients in China (N = 1190; CCT: 539.2 ± 37.8 μm) (34). However, ECD was not reported in this study. Similarly, a study on Thai individuals, who also share an Asian ethnic background, reported CCT and ECD values in healthy participants of the same age group that are closely aligned with our results (35). In contrast, the CCT and ECD of Egyptians aged 20–30 years (CCT: 525.92 ± 46.83 μm; ECD: 2933.75 ± 345.92 cells/mm²) and 30–40 years (CCT: 518.65 ± 67.99 μm; ECD: 2693.57 ± 287.34 cells/mm²) revealed significantly lower values compared to our findings (24). Of interest, a study on young Turkish individuals (20–40 years) reported CCT values (550.3 ± 34.6 μm) that are generally consistent with ours, whereas their ECD values (2659 ± 283 cells/mm²) were found to be significantly lower (36).

Our findings revealed no statistically significant gender-based differences in ECD (ANCOVA, adjusting for age). However, male participants demonstrated significantly higher CCT measurements compared to females (550.34 ± 29.05 μm vs. 543.62 ± 28.57 μm; *P* = 0.031; ANCOVA, adjusting for age). These results are consistent with a previous study conducted on young myopia patients in China (34). Several studies have similarly reported no statistically significant gender-based differences in ECD or CCT among healthy populations in Egypt (24), Israel (37), and Sudan (21). Intriguingly, two population-based studies in Japan presented conflicting findings regarding ECD: one study reported higher endothelial cell density in males (38), while the other found superior values in females (22). Consequently, the current body of evidence remains inconclusive in identifying definitive gender-dependent variations in CCT or ECD. Previous studies have documented that male eyes are typically larger in overall dimensions, correlating with greater body stature and orbital development (39). Hormonal influences, particularly androgen-mediated differences in collagen synthesis and corneal biomechanics, may contribute to thicker corneas in males. Additionally, differences in lifestyle factors, occupational exposures, and possibly unmeasured variables such as hormonal status could influence these parameters (40).

This study has several limitations that warrant acknowledgment. First, the research employed a single-center, cross-sectional design with a relatively small sample size, which may restrict the generalizability of the findings. The single-center aspect could also introduce selection bias, as the characteristics of participants might reflect local demographic nuances rather than representing a broader population. Second, the study primarily concentrated on Chinese myopic adults aged 18 to 43 years. As a result, the corneal parameters observed may not accurately reflect other refractive status groups (such as hyperopes or emmetropes) or populations of differing ethnic backgrounds. Third, a major limitation was the inability to accurately document and analyze the specific duration of contact lens wear among participants. Previous research has shown that prolonged contact lens use can induce morphological changes in the cornea, highlighting the importance of this factor, which was unfortunately omitted from precise evaluation in this study. Lastly, a critical assessment in candidates for keratorefractive surgery is the determination of whether they may have suspicious or confirmatory signs of corneal ectasia, which is generally evaluated using corneal tomographers. In various studies, it has been found that between 4.1% and 17.5% of candidates for excimer laser refractive surgery are diagnosed with suspected keratoconus or keratoconus (41–43). In our study, 5.0% (20/400) of eyes showed thin pachymetry (less than 500 microns). Unfortunately, the database did not capture which of those eyes had tomographic signs of keratoconus (Pentacam HR), which also constitutes a limitation of the present research.

In conclusion, refractive errors and axial elongation do not significantly affect CCT, corneal endothelial cell density, or cell morphology in our cohort of refractive surgery candidates. However, age demonstrates a mild correlation with both CCT and ECD in this study group, which spans nearly two decades. Additionally, the potential influence of gender and ocular laterality on corneal parameters warrants careful assessment during preoperative evaluations.

## Data Availability

The original contributions presented in the study are included in the article/supplementary material. Further inquiries can be directed to the corresponding author.

## References

[1] Liang J Pu Y Chen J Liu M Ouyang B Jin Z . Global prevalence, trend and projection of myopia in children and adolescents from 1990 to 2050: a comprehensive systematic review and meta-analysis. Br J Ophthalmol. (2025) 109:362–71. doi: 10.1136/bjo-2024-325427, PMID: 39317432

[2] Lawrenson JG Huntjens B Virgili G Ng S Dhakal R Downie LE . Interventions for myopia control in children: a living systematic review and network meta-analysis. Cochrane Database Syst Rev. (2025) 2:CD014758. doi: 10.1002/14651858.CD014758.pub3, PMID: 39945354 PMC11822883

[3] Galvis V Villamil JF Acuña MF Camacho PA Merayo-Lloves J Tello A . Long-term endothelial cell loss with the iris-claw intraocular phakic lenses (Artisan^®^). Graefe’s Arch Clin Exp Ophthalmol. (2019) 257:2775–87. doi: 10.1007/s00417-019-04506-9, PMID: 31659458

[4] Li J Song LL Song H . Five-year clinical outcomes of rigid iris-fixated phakic intraocular lens in northern Chinese. Int Ophthalmol. (2022) 42:2551–61. doi: 10.1007/s10792-022-02303-8, PMID: 35381897

[5] Kalra N Asif MI Bafna RK Sharma N Sinha R . Posterior chamber phakic intraocular lens implantation for refractive correction in corneal ectatic disorders: A review. J Refract Surg. (2021) 37:351–9. doi: 10.3928/1081597X-20210115-03, PMID: 34044697

[6] Juda M Bedlinski M Roszkowska AM Wierzbowska J . Clinical evaluation of corneal endothelial parameters following laser refractive surgery in myopic eyes: A review. J Clin Med. (2024) 13:1665. doi: 10.3390/jcm13061665, PMID: 38541890 PMC10971698

[7] Vroman DT Solomon KD Holzer MP Peng Q Apple DJ Bowie EM . Endothelial decompensation after laser in *situ* keratomileusis. J Cataract Refract Surg. (2002) 28:2045–9. doi: 10.1016/s0886-3350(01)01352-9, PMID: 12457684

[8] Dastjerdi MH Sugar A . Corneal decompensation after laser in *situ* keratomileusis in fuchs’ endothelial dystrophy. Cornea. (2003) 22:379–81. doi: 10.1097/00003226-200305000-00020, PMID: 12792486

[9] Moshirfar M Feiz V Feilmeier MR Kang PC . Laser in *situ* keratomileusis in patients with corneal guttata and family history of Fuchs’ endothelial dystrophy. J Cataract Refract Surg. (2005) 31:2281–6. doi: 10.1016/j.jcrs.2004.05.061, PMID: 16473218

[10] Kisiel FB Gurumurthy GJ . Endothelial cell loss post-implantable collamer lens V4c: meta-analysis. J Cataract Refract Surg. (2024) 50:420–3. doi: 10.1097/j.jcrs.0000000000001389, PMID: 38194352

[11] Wang Z Zuo X Liu L Chen X Li R Zhu H . Corneal endothelial cell density and its correlation with birth weight, anthropometric parameters, and ocular biometric parameters in Chinese school children. BMC Ophthalmol. (2022) 22:334. doi: 10.1186/s12886-022-02561-1, PMID: 35933331 PMC9356483

[12] Norhani M Low YC Bariah MA Mizhanim MS Norlaili A . Corneal endothelial morphology of healthy myopic Malaysian children of Chinese ethnicity aged 8–9 years and its association with axial length. F1000Res. (2022) 11:339. doi: 10.12688/f1000research.110560.2, PMID: 36111215 PMC9459173

[13] Liang H Zuo HY Chen JM Cai J Qin YZ Huang YP . Corneal endothelial cell density and morphology and central corneal thickness in Guangxi Maonan and Han adolescent students of China. Int J Ophthalmol. (2015) 8:608–11. doi: 10.3980/j.issn.2222-3959.2015.03.31, PMID: 26086017 PMC4458672

[14] Li Y Fu Z Liu J Li M Zhang Y Wu X . Corneal endothelial characteristics, central corneal thickness, and intraocular pressure in a population of chinese age-related cataract patients. J Ophthalmol. (2017) 2017:9154626. doi: 10.1155/2017/9154626, PMID: 28630766 PMC5463134

[15] Miao AO Lin P Qian D Xu J Lu YI Zheng T . Association between endothelial cell density and corneal thickness in medium, short, and long eyes of han chinese cataract patients. Am J Ophthalmol. (2024) 262:10–8. doi: 10.1016/j.ajo.2024.01.036, PMID: 38316200

[16] Zhang Y Zhang S Zhang K Lu Y Zhu X . Characteristics of the corneal endothelium in elderly adults with high myopia. Phenomics. (2024) 4:562–9. doi: 10.1007/s43657-024-00186-6, PMID: 40061825 PMC11889312

[17] Da Silva F Linhares JMM Lira M . What intrinsic factors affect the central corneal thickness? Ophthalmic Physiol Opt. (2025) 45:315–32. doi: 10.1111/opo.13414, PMID: 39495112

[18] Panda L Mohapatra S Khuntia I . A cross-sectional study on central corneal thickness in relation to age, gender, refractive errors, and axial length among patients visiting tertiary care center in south India. Eur J Cardiovasc Med. (2025) 15:200–4. doi: 10.5083/ejcm/25-03-34, PMID: 36468563

[19] Hamza MN Roshdy MM Seleet MM El Raggal TM . Correlation between ocular biometric parameters and corneal endothelium in a sample of young Egyptian adults. Med Hypothesis Discov Innov Ophthalmol. (2021) 10:121–8. doi: 10.51329/mehdiophthal1430, PMID: 37641708 PMC10460222

[20] Hafeez T Alvi K Yousaf S Ashraf MA Shahid S Hussain K . Correlation of corneal endothelial cell density in low, moderate, and higher myopes. Rawal Med J. (2023) 48:957–60.

[21] Mutwaly RF Alrasheed SH Elmadina AEM Aldakhil S . Morphology and thickness of corneal endothelial cells in young Sudanese individuals with myopia. J Med Life. (2023) 16:1808–12. doi: 10.25122/jml-2023-0251, PMID: 38585539 PMC10994625

[22] Ono T Mori Y Nejima R Iwasaki T Miyai T Miyata K . Corneal endothelial cell density and morphology in ophthalmologically healthy young individuals in Japan: An observational study of 16842 eyes. Sci Rep. (2021) 11:18224. doi: 10.1038/s41598-021-97776-5, PMID: 34521951 PMC8440503

[23] Jonas JB Jonas RA Bikbov MM Wang YX Panda-Jonas S . Myopia: Histology, clinical features, and potential implications for the etiology of axial elongation. Prog Retin Eye Res. (2023) 96:101156. doi: 10.1016/j.preteyeres.2022.101156, PMID: 36585290

[24] Abdellah MM Ammar HG Anbar M Mostafa EM Farouk MM Sayed K . Corneal endothelial cell density and morphology in healthy Egyptian eyes. J Ophthalmol. (2019) 2019:6370241. doi: 10.1155/2019/6370241, PMID: 30918718 PMC6409007

[25] Shaikh A Paracha Q Sarwar MI . Relationship of Corneal Endothelial Morphology and Central Corneal Thickness to Age and Sex in Normal Healthy individuals in our Community. J Sheikh Zayed Med Coll (JSZMC). (2022) 13:18. doi: 10.47883/jszmc.v13i3.217

[26] Xu G Wu G Du Z Zhu S Guo Y Yu H . Distribution of white-to-white corneal diameter and anterior chamber depth in chinese myopic patients. Front Med (Lausanne). (2021) 8:732719. doi: 10.3389/fmed.2021.732719, PMID: 34869427 PMC8639187

[27] Prada AM Quintero F Mendoza K Galvis V Tello A Romero LA . Assessing fuchs corneal endothelial dystrophy using artificial intelligence-derived morphometric parameters from specular microscopy images. Cornea. (2024) 43:1080–7. doi: 10.1097/ICO.0000000000003460, PMID: 38334475 PMC11296282

[28] Ono T Sakisaka T Takada K Tokuda S Mori Y Nejima R . Long-term effect of using hard contact lenses on corneal endothelial cell density and morphology in ophthalmologically healthy individuals in Japan. Sci Rep. (2023) 13:7649. doi: 10.1038/s41598-023-34756-x, PMID: 37169893 PMC10175498

[29] Ono T Kaida T Higashi S Mori Y Nejima R Iwasaki T . Corneal endothelial density and morphology in long-term soft contact lens users in Japan: a retrospective cross-sectional study of 17,732 eyes. Cutan Ocul Toxicol. (2024) 43:335–40. doi: 10.1080/15569527.2024.2408685, PMID: 39352078

[30] Mutwaly RF Sr . Corneal endothelial polymegathism and pleomorphism induced by daily-wear soft contact lenses. Cureus. (2024) 16:e74187. doi: 10.7759/cureus.74187, PMID: 39583607 PMC11582090

[31] Xu G Hu Y Zhu S Guo Y Xiong L Fang X . A multicenter study of interocular symmetry of corneal biometrics in Chinese myopic patients. Sci Rep. (2021) 11:5536. doi: 10.1038/s41598-021-84937-9, PMID: 33692402 PMC7946893

[32] Bikbov MM Gilmanshin TR Zainullin RM Kazakbaeva GM Zaynetdinov AF Nuriev IF . Central corneal thickness and its associations in a Russian population. The Ural eye and Medical Study. Eye (Lond). (2023) 37:705–13. doi: 10.1038/s41433-022-02026-1, PMID: 35347290 PMC9998395

[33] Kelekele JT Kayembe Lubeji DL Mwanza JC . Interocular symmetry and repeatability of central corneal thickness and corneal endothelial cell morphology and density in healthy eyes of congolese. Semin Ophthalmol. (2022) 37:241–8. doi: 10.1080/08820538.2021.1974497, PMID: 34543164

[34] Wang Q Liu W Wu Y Ma Y Zhao G . Central corneal thickness and its relationship to ocular parameters in young adult myopic eyes. Clin Exp Optom. (2017) 100:250–4. doi: 10.1111/cxo.12485, PMID: 27757993

[35] Tananuvat N Khumchoo N . Corneal thickness and endothelial morphology in Normal Thai eyes. BMC Ophthalmol. (2020) 20:167. doi: 10.1186/s12886-020-01385-1, PMID: 32345246 PMC7187506

[36] Furundaoturan O Selver OB Palamar M . Evaluation of corneal endothelial cell loss peak in young healthy turkish population. Ann Ophthalmol Vis Sci. (2022) 5:1022.

[37] Mimouni M Flores V Shapira Y Graffi S Levartovsky S Sela T . Correlation between central corneal thickness and myopia. Int Ophthalmol. (2018) 38:2547–51. doi: 10.1007/s10792-017-0766-1, PMID: 29075941

[38] Aketa N Uchino M Kawashima M Uchino Y Yuki K Ozawa Y . Myopia, corneal endothelial cell density and morphology in a Japanese population-based cross-sectional study: the JPHC-NEXT Eye Study. Sci Rep. (2021) 11:6366. doi: 10.1038/s41598-021-85617-4, PMID: 33737603 PMC7973534

[39] Kolačko Š Predović J Kokot A Bosnar D Brzović-Šarić V Šarić B . Do gender, age, body mass and height influence eye biometrical properties in young adults? A pilot study. Int J Environ Res Public Health. (2021) 18:11719. doi: 10.3390/ijerph182111719, PMID: 34770229 PMC8582935

[40] Komninou MA Seiler TG Enzmann V . Corneal biomechanics and diagnostics: a review. Int Ophthalmol. (2024) 44:132. doi: 10.1007/s10792-024-03057-1, PMID: 38478103 PMC10937779

[41] Galvis V Tello A Jaramillo J Gutierrez A Rodriguez L Quintero M . Prevalence of keratoconus patients who consulted with a desire refractive surgery in ophthalmology center reference Bucaramanga, Colombia. Rev Soc Colomb Oftal. (2011) 44:129–34.

[42] Saro AS Radwan GA Mohammed UA Abozaid MA . Screening for keratoconus in a refractive surgery population of Upper Egypt. Delta J Ophthalmol. (2018) 19:19–23. doi: 10.4103/DJO.DJO_39_17

[43] Abdelghany AA Omar I . Analysis of topographic corneal parameters in a large cohort of corneal refractive surgery candidates. Saudi J Ophthalmol. (2025). doi: 10.4103/sjopt.sjopt_237_24 PMC13082547 41994243

